# Mitochondria-Mediated Apoptosis and Autophagy Participate in Buprofezin-Induced Toxic Effects in Non-Target A549 Cells

**DOI:** 10.3390/toxics10100551

**Published:** 2022-09-21

**Authors:** Yuanhang Ren, Xuan He, Yanting Yang, Yanan Cao, Qiang Li, Lidan Lu, Lianxin Peng, Liang Zou

**Affiliations:** 1 Key Laboratory of Coarse Cereal Processing, Ministry of Agriculture and Rural Affairs, Chengdu 610106, China; 2 Sichuan Engineering and Technology Research Center of Coarse Cereal Industralization, Chengdu 610106, China; 3 College of Food and Biological Engineering, Chengdu University, Chengdu 610106, China

**Keywords:** pesticide, mitochondrial dysfunction, programmed cell death, transcriptome

## Abstract

Buprofezin (BUP) is an insecticide used for control of sucking pests. Its widespread use has raised concerns about possible adverse effects on the environment, and especially human health. The mechanism of toxicity of BUP, with respect to human health, is still unclear. Consequently, human A549 cells were employed to clarify the cytotoxicity and toxic mechanism of BUP at the molecular and cellular levels. The outcomes revealed BUP latent toxicity to A549 in a time- and dose-related way. Moreover, BUP induced mitochondrial dysfunction associated with mitochondrial membrane potential collapse, mitochondrial calcium overload, and ROS aggregation, ultimately resulting in the apoptosis and autophagy of A549 cells. Symbolic apoptotic and autophagic modifications were detected, including leakage of cyt-c, elevation of Bax/Bcl-2, activation of cas-9/-3, constitution of autophagic vacuoles, promotion of Beclin-1, conversion of LC3-II, and reduction of p62. Additionally, in total, 1216 differentially expressed genes (DEGs) were defined after BUP treatment. Several apoptosis- and autophagy-related genes, such as BCL2, ATG5, and ATG16, down- or upregulated at the RNA transcription level, and functional DEGs enrichment analysis showed their involvement in the metabolism of xenobiotics by cytochrome P450, mTOR signalling pathway, and AMPK signalling pathway. Results confirmed that BUP could induce cytotoxicity associated with mitochondria-mediated programmed cell death in A549 cells.

## 1. Introduction

To guarantee food production, agricultural chemicals are universally employed to prevent and control pests and plant pathogens. However, due to the degradation and enrichment features of some pesticides, the latent adverse impacts on non-targeted organisms, including humans, have become a major concern [1,2,3]. As reported by the World Health Organization (WHO), pesticide poisoning results in approximately 0.2 million deaths per year worldwide [4,5]. Therefore, taking into account the environmental and health risks, many high-efficiency and low-toxicity pesticides, such as insect growth regulator insecticides, have been developed and replaced some traditional pesticides. Buprofezin (BUP) is a type of insect growth regulator insecticide and is different from other nerve- or stomach-toxic insecticides. BUP acts as an inhibitor of chitin synthesis and molting, which interferes with the normal development of insects, ultimately leading to death. The suspending agent of buprofezin (25% SC) is widely used to control sucking pests of the orders Hemiptera and Thysanoptera, such as leafhoppers, plant hoppers, jassids, thrips, whiteflies, aphids, mealy bugs, etc. [6,7,8].

BUP is highly stable under alkaline and acidic conditions, and can be readily adsorbed by soil and remain in it virtually indefinitely. Under aerobic conditions, the BUP half-life is nearly 60 days, on average, while under anaerobic conditions, such as flooded field areas, its half-life increases to more than 100 days [9,10]. Although BUP is generally supposed to be safe for non-target organisms, its characteristic persistence might become a potential health hazard. Previous studies have confirmed that BUP residues can be detected in crops, fruits, vegetables, soil, and water ecosystems [11,12]. In the water environment, even at low concentrations, BUP could be detrimental to African catfish early embryonic stages and inhibit larval growth [13]. However, adverse effects on mammals have rarely been revealed. Only a few studies have indicated that sublethal doses induce hepatotoxicity in mice by promoting energy metabolism alteration from oxidative phosphorylation to anaerobic glycolysis [4,14]. Additionally, BUP exposure significantly increases the prevalence of micronuclei in hamster embryos [15].

In consideration of the widespread use of BUP and its potential for being a health hazard, it is essential to reassess its toxicity and further investigate its effects on humans at the cellular and molecular levels. Therefore, in this paper, the frequently used human cellular model A549 was used to assess BUP cytotoxicity and reveal its mode of action in humans. We found that BUP could inhibit A549 cell viability related to mitochondria-intervened programmed cell death (PCD). These findings highlight the potential threats to human health posed by BUP. Additionally, this study describes the toxic mechanisms of BUP in humans, which may inform the treatment of BUP poisoning.

## 2. Materials and Methods

### 2.1. Chemicals

Buprofezin (BUP, purity of 98%) was obtained from MOLBASE (Shanghai, China). BUP stock solution was dissolved in dimethyl sulfoxide (DMSO) (MP Biomedicals, Santa Ana, CA, USA) and kept at 4 °C. Culture medium was used to dilute the stock solution to the work solution with various concentrations and to ensure DMSO concentration lower than 0.1%.

### 2.2. Cell Lines and Culture

The A549 cell line was acquired from the China Center for Typical Culture Collection (CCTCC, Wuhan, China). The cells were cultured in Roswell Park Memorial Institute (RPMI-1640) medium (Sigma, St. Louis, MO, USA) supplemented with 10% fetal bovine serum (Tianhang, Hangzhou, China), 100 units/mL penicillin, and 100 mg/mL streptomycin (Gibco, NY, USA). The cells were maintained in a 100 mm diameter cell culture dish (BIOFIL, Guangzhou, China) at a humidified atmosphere (37 °C, 5% CO_2_), and passaged twice in one week [16].

### 2.3. Cytotoxicity Assay

The thiazolyl blue tetrazolium bromide (MTT) assay was used to evaluate the cytotoxicity of BUP to A549 cells [17]. The 100 μL medium with 1 × 10^4^ cells was seeded into each well of 96-well microplates (BIOFIL, Guangzhou, China). After 12 h, 10, 20, 40, 80, and 160 μM BUP was added into the wells for 24, 48, and 72 h, respectively. Cell proliferation inhibition rate was calculated as the percentage of absorbance in the control (0.1% DMSO-treated cells). Calculation formula: (OD_control_ − OD_treat_)/OD_control_ × 100%.

### 2.4. Apoptosis Analysis

Apoptotic cells were detected by Annexin V and PI staining assay [18]. The 2 mL medium with 2 × 10^5^ cells were seeded into a petri dish. Then, the cells were exposed to BUP at concentrations of 20 μM (0.5 × IC_50_), 40 μM (IC_50_), 60 μM (1.5 × IC_50_), and 80 μM (2 × IC_50_) for 48 h. The 0.1% DMSO-treated cells served as a control. Cells were collected by centrifuge (2000× *g*, 3 min) and washed two times with phosphate-buffered saline (PBS) (Servicebio, Wuhan, China). Then cells were stained with Annexin V and PI (Keygen, Nanjing, China) for 15 min, respectively. Lastly, cells were measured by means of flow cytometry (BD CantoⅡ, NJ, USA), and the percentage of apoptotic cells was calculated with FlowJo (V10, 2015) software (BD, Ashland, OR, USA).

### 2.5. Mitochondrial Membrane Potential Analysis

A JC-1 staining assay was utilized to quantify the mitochondrial transmembrane potential (MMP) [19]. The 2 mL medium with 2 × 10^5^ cells was seeded into a petri dish and exposed to BUP for 48 h at 20, 40, 60, and 80 μM. The 0.1% DMSO-treated cells were employed as a control. Then, cells were incubated with 2 μM JC-1 (Keygen, Nanjing, China) for 20 min at 37 °C. Then, cells were collected by means of centrifuge (2000× *g* for 3 min) and washed two times with PBS. The fluorescence intensities of the JC-1 monomer (green) and JC-1 aggregate (red) were analysed with flow cytometry (BD CantoⅡ, Franklin Lakes, NJ, USA). The statistics of the loss of MMP were calculated using the declining level of red fluorescence vs. control using FlowJo (V10, 2015) software (BD, Ashland, OR, USA).

### 2.6. Western Blot Analysis

The 2 mL medium with 2 × 10^5^ cells was seeded into a petri dish and exposed to BUP for 48 h at 20, 40, 60, and 80 μM. Cells treated with 0.1% DMSO served as a control. A cell mitochondria isolation kit (Beyotime, Shanghai, China) was used to collect mitochondria separately. Mitochondrial proteins and total proteins were extracted by cold radio-immunoprecipitation assay (RIPA) lysis buffer (Beyotime, Shanghai, China) with 1 mM phenylmethylsulfonyl fluoride (PMSF) (Beyotime, Shanghai, China). Moreover, protein dose was measured with a bicinchoninic acid (BCA) protein assay kit (Beyotime, Shanghai, China). The protein samples (30 μg) were separated by 15% SDS-PAGE (Beyotime, Shanghai, China) and transferred to PVDF (Millipore, MA, USA) membranes by electrophoresis. The blots were blocked in Tris-Tween (Beyotime, Shanghai, China) with 5% non-fat dry milk powder at room temperature for 1 h. Then the blots were incubated with primary antibodies (LC-3, cyt-c, p62, Bcl-2, Beclin-1, Bax, beta-actin, and VDAC1) and HRP-conjugated secondary antibodies (Servicebio, Wuhan, China). The immune-reactive proteins were imagined using a SuperSignal kit (Pierce, IL, USA). The gray band values were quantified by ImageLab (V3, 2010) software (BioRad, Hercules, CA, USA). The results showed the ratios of protein intensity in treated cells and the corresponding proteins in control cells [20].

### 2.7. Caspase-9/-3 Activity Analysis

The 2 mL medium with 2 × 10^5^ cells was seeded into a petri dish and exposed to BUP for distinct time periods (6, 12, and 24 h) at 80 μM. The 0.1% DMSO- treated cells were used as a control. Caspase activity was evaluated using caspase-3 and -9 assay kits (Keygen, Nanjing, China). Cells were collected by centrifuge (10,000× *g*, 10 min, 4 °C) and resuspended in lysis buffer, and subsequently hatched on ice for 1 h. The 50 μL supernatant was mixed with 50 μL reaction buffer and 5 μL caspase-3 or -9 substrate, then hatched at 37 °C for 4 h. Lastly, caspase-3 and caspase-9 activities were measured at 405 nm with a microplate reader (BioTek, VT, USA) [21].

### 2.8. Autophagy Analysis

The autophagic ultrastructure was observed using transmission electron microscopy (TEM) (JEOL, Tokyo, Japan). Cells were exposed to BUP for 48 h at distinct dose (40, 60, and 80 μM). Cells treated with 0.1% DMSO served as a control. Cells were collected and washed two times with PBS, and then fixed in 2.5% glutaraldehyde (Macklin, Shanghai, China) at 4 °C for 12 h. After being washed two times with PBS, the cells were postfixed in 1% osmium tetroxide (Macklin, Shanghai, China) for 45 min and then washed two times with the same buffer. The fixed cells were dried using graded acetone and embedded in Epon 812 (Fluka, Buchs, Switzerland). Ultrathin sections were dyed with lead citrate and uranyl acetate (Macklin, Shanghai, China), then observed using a transmission electron microscope [22,23]. The constitution of the autophagosomes was detected by monodansylcadaverine (MDC) (Beyotime, Shanghai, China) dyes. Cells were hatched with 1 μg/mL MDC in the dark for 30 min and then washed twice with PBS before watched by fluorescence microscope (Leica, Wetzlar, Germany) [23].

### 2.9. Reactive Oxygen Species Analysis

Cells were treated with BUP for 48 h at distinct doses (40, 60, and 80 μM). The 0.1% DMSO-treated cells were used as a control. Then, the DCFH-DA (Keygen, Nanjing, China) was used to analyse intracellular ROS generation [23]. Cells were harvested by centrifuge (2000× *g*, 3 min) and washed two times with PBS, then hatched with 10 μM DCFH-DA for 30 min at 37 °C. The DCFH-DA was hydrolysed to DCFH and then oxidized to DCF. Its green fluorescence was detected using flow cytometry (BD CantoⅡ, Franklin Lakes, NJ, USA). The statistics of increased ROS level were calculated using the DCF fluorescence-positive cell ratio by FlowJo (V10, 2015) software (BD, Ashland, OR, USA).

### 2.10. Cytosolic and Mitochondrial Ca^2+^ Levels Analysis

Cells were exposed to distinct doses of BUP (40, 60, and 80 μM) for 48 h. Cells exposed to 0.1% DMSO served as a control. The collected cells were hatched with 1 μM Fluo-3AM and 2 μM Rhod-2AM for 30 min at 37 °C. Then, the stained cells were washed with Hanks’ buffer (Beyotime, Shanghai, China) and further hatched with PBS for 30 min at 37 °C before being detected with a flow cytometer (BD CantoⅡ, Franklin Lakes, NJ, USA). The increased Ca^2+^ level was statistically analysed using the green and red fluorescence intensities by FlowJo (V10, 2015) software (BD, Ashland, OR, USA) [23].

### 2.11. RNA Sequencing Analysis

Cells were exposed to 80 μM BUP for 48 h. Cells exposed to 0.1% DMSO served as a control. The methods of RNA extraction, purification, concentration, and integrity detection are detailed in previous reports [24]. The cDNA library and sequence building was performed with an RNA sample prep kit (Illumina, San Diego, CA, USA). The dsDNA system (QuantiFluor, Promega) and bridge polymerase chain reaction (PCR) were used to quantify the cDNA library and generate clusters. Lastly, the HiSeq X Ten platform (Illumina, San Diego, CA, USA) applied to the complete sequence.

SeqPrep and Sickle were applied as the quality and statistics control of the original sequencing data. Then, TopHat was utilized to compare clean reads with the reference genome. While, RSeQC-2.3.6 was employed to assessed the quality of the sequencing comparison results.

To determine the differentially expressed genes (DEGs) in distinct treatments, each transcript’s expression level was calculated according to FRKM (fragments per kilobase of exon per million fragments mapped). RESM (reads per kilobase of exon model per million mapped reads) was used to quantify gene expression. The DESeq2 software was applied to the different analyzed expressions. The *p*-adjust < 0.05 and |log2FC| ≥ 1 was set as screening conditions. The function enrichment analyses were performed by the BLASTX algorithm, and every DEG was compared with data from the Kyoto Encyclopedia of Genes and Genomes (KEGG), National Center for Biotechnology Information (NCBI), non-redundant (NR) protein sequence database, and Gene Ontology (GO) database. Then, KEGG and GO enrichment were analyzed using KOBAS and GOATOOLS, respectively.

### 2.12. Statistics

The assays in this research were performed at least three times. The statistical analyses were performed using Excel 2019 and SPSS v18. The data were shown as the means ± standard errors of the mean (SEMs). Mathematical significance was defined using ANOVA and Student’s *t*-tests (** *p* ≤ 0.01, * *p* ≤ 0.05).

## 3. Results

### 3.1. Cytotoxic Effects of BUP on A549 Cells

**Figure 1:**
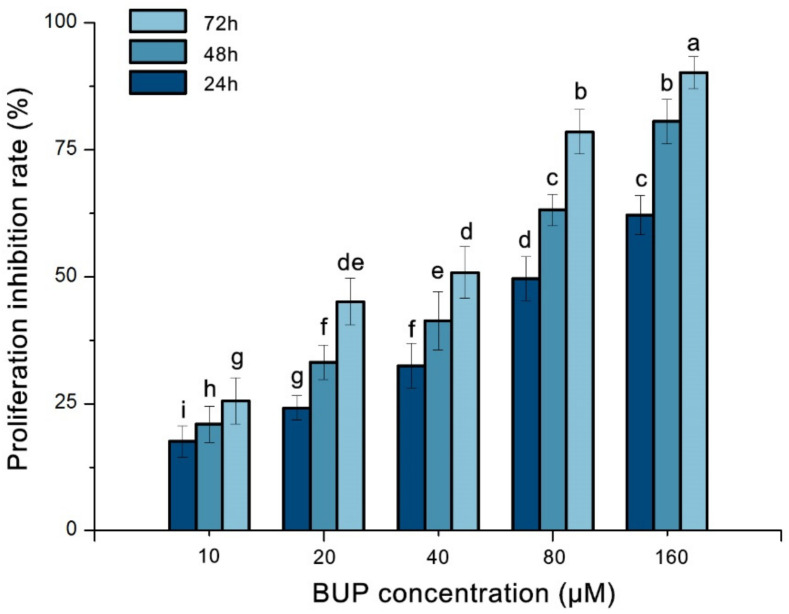
Cytoactivity of buprofezin (BUP) against A549 cells at various concentrations for various lengths of time. Different lowercase letters above columns indicate statistical differences from each other at *p* ≤ 0.05. Columns with the same letters are not significantly different.

The results displayed in Figure 1 indicate that BUP inhibited A549 proliferation in a time- and dose-related way. The proliferative inhibition rates of A549 cells at 24 h were 17.53 ± 2.9%, 24.16 ± 2.6%, 32.46 ± 3.9%, 49.58 ± 3.8%, and 62.12 ± 4.1% after treatment with BUP concentrations of 10, 20, 40, 80, and 160 μM, respectively. Of note, the IC_50_ of BUP treatments at 24, 48, and 72 h were 87.45, 43.77, and 27.36 μM, respectively.

### 3.2. BUP-Induced Apoptosis in A549 Cells

**Figure 2:**
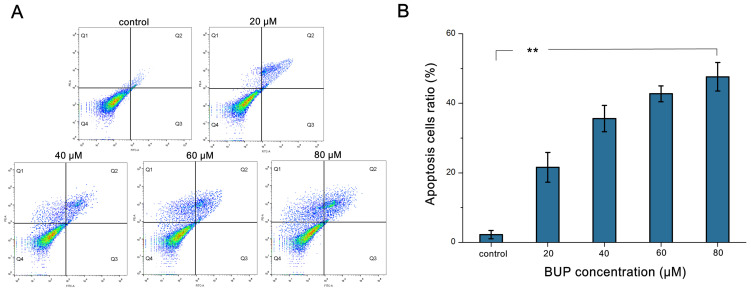
Analysis of apoptotic A549 cells after treatment with BUP at various concentrations, showing: (**A**) investigation of annexin V and PI dyeing by flow cytometry, (**B**) measurement of apoptotic cells. ** *p* ≤ 0.01 vs. negative control.

As displayed in Figure 2A, the amount of early apoptotic cells and apoptotic cells grew markedly after exposure to distinct doses of BUP within concentrations from 0 to 80 μM. In comparison to the control (2.21 ± 1.2%), the apoptotic cells increased remarkably to 47.62 ± 4.1% after treatment with BUP at 80 μM (Figure 2B). The outcomes clearly stated that BUP induced apoptosis of A549 cells in a dose-related way.

**Figure 3:**
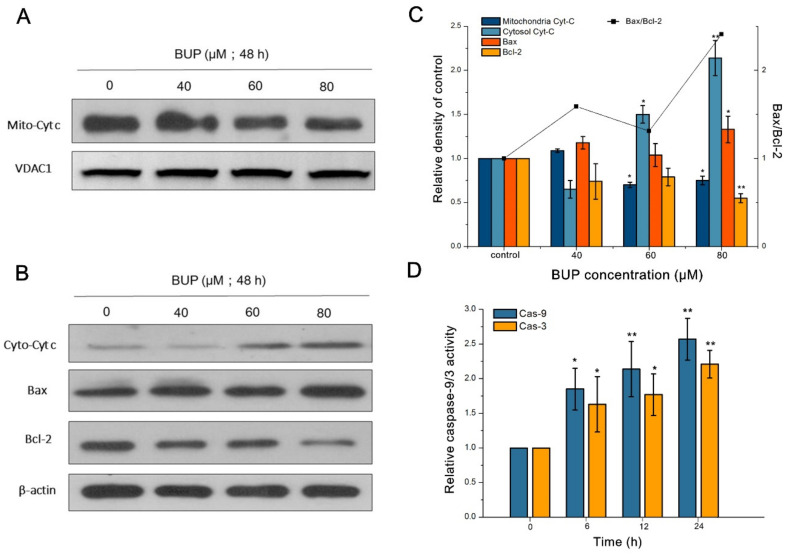
Investigation of apoptotic protein expression in A549 cells after exposure to BUP at different concentrations and for various time intervals, showing: (**A**,**B**) results of cyt-c distribution, Bcl-2, and Bax in A549 cells by western blot, (**C**) intensity of Bax/Bcl-2 and cyt-c ratio, and (**D**) activity of caspase-3 and -9. * *p* ≤ 0.05 and ** *p* ≤ 0.01 vs. negative control.

As displayed in Figure 3A,B, cytochrome c (cyt-c) decreased in the mitochondrial fractions in a dose-related way following BUP treatment. By contrast, the aggregation of cyt-c was observed in the cytosolic fractions. Moreover, the results showed that BUP induced a decrease in the percentage of apoptotic proteins Bcl-2/Bax in a concentration-related way, with an increase in Bax and a decrease in Bcl-2. Whether cell apoptosis was involved in caspase activation was further examined. Caspase-3 and its precursor, caspase-9, were activated by BUP in a dose-related way (Figure 3C).

### 3.3. Production of ROS and Mitochondrial Damage in BUP-Treated Cells

**Figure 4:**
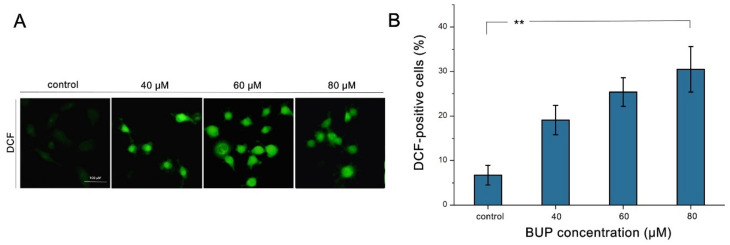
Analysis of ROS production in A549 cells after treatment with BUP at various concentrations, showing: (**A**) observation of DCF dyeing by epifluorescence (200×), (**B**) quantification of ROS levels. ** *p* ≤ 0.01 vs. negative control.

As demonstrated in Figure 4A, a gradual strengthening of green fluorescence was observed in cells with increase in BUP dose. Additionally, a significant increase in green fluorescence (30.5 ± 4.8%) was found in 80 μM BUP-treated cells (Figure 4B), compared with the control. The results suggest that BUP could induce the generation of ROS in A549 cells.

**Figure 5:**
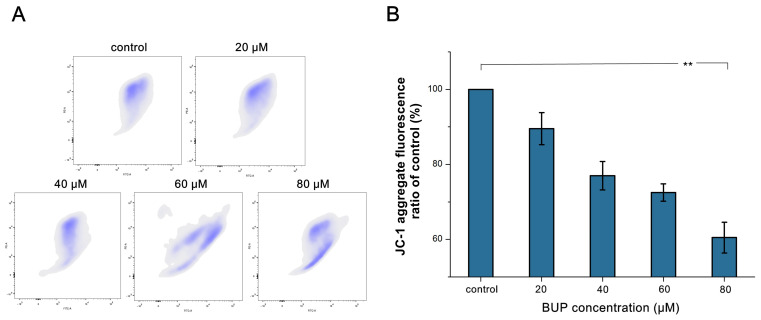
Investigation of MMP decline in A549 cells after exposure to BUP at various concentrations, showing: (**A**) detection of JC-1 dyeing by flow cytometry, (**B**) quantification of MMP decline levels. ** *p* ≤ 0.01 vs. negative control.

Overdose of ROS could lead to injury of mitochondrial cellular membranes and ultimately cause mitochondrial damage [25]. As revealed in Figure 5, treatment with 80 μM BUP resulted in a 39.52 ± 3.9% decrease in the mitochondrial membrane potential (MMP) compared with control. The dose-related decline in JC-1 aggregate fluorescence intensity proved the depolarization of the MMP.

**Figure 6:**
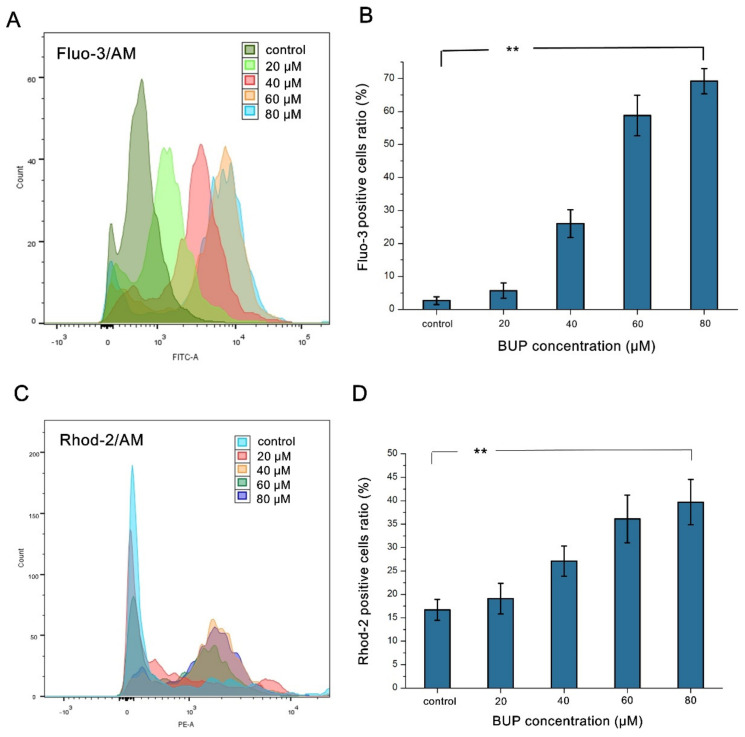
Analysis of cytosolic and mitochondrial Ca2+ levels in BUP-treated A549 cells, showing: (**A**,**C**) analysis of fluo-3 (**A**) and rhod-2 (**C**) dyeing by flow cytometry, (**B**) measurement of fluo-3 fluorescence intensity, (**D**) measurement of rhod-2 fluorescence intensity. ** *p* ≤ 0.01 vs. negative control.

As MMP collapse and cell death may be indirectly stimulated by mitochondrial matrix Ca^2+^ overload [26], we then examined the variation of Ca^2+^ in the mitochondria and cytosol utilizing mitochondrial Ca^2+^ indicator dye (Rhod-2AM) and cytoplasmic Ca^2+^ indicator dye (Fluo-3AM). Figure 6 illustrates that BUP increased Ca^2+^ levels via a dose-related manner in both the mitochondria and the cytoplasm. The results further confirm that BUP disturbed intracellular calcium homeostasis in A549 cells.

### 3.4. BUP-Induced Autophagy in A549 Cells

**Figure 7:**
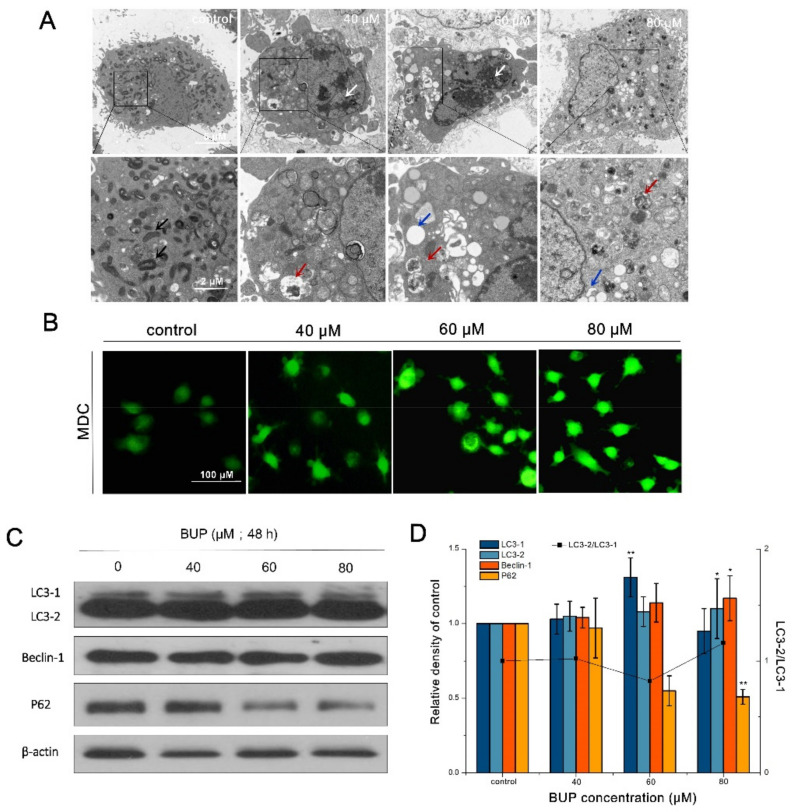
Investigation of autophagic effects of A549 cells after exposure to BUP at different concentrations, showing: (**A**) observation of autophagic submicrostructure via TEM, normal cells with mitochondria in elliptical shape (black arrows), BUP-treated cells with autophagic vacuoles (blue arrows), autophagic vacuoles with content (red arrows) and chromatin condensation (white arrows); (**B**) observation of MDC dyeing by epifluorescence (200×); (**C**) detection of autophagy-related proteins; (**D**) expression of autophagy-related proteins. * *p* ≤ 0.05 and ** *p* ≤ 0.01 vs. the negative control.

TEM assays were used for observing the characteristics of autophagy. Figure 7A shows the normal cells with an ordinary dense and uniform cytoplasm. However, great autophagic vacuoles containing cellular material were observed in A549 cells, after exposure to BUP. Furthermore, MDC, a fluorescent dye for autophagy monitoring, was applied to observe the formation of autophagosomes. As revealed in Figure 7B, it was seen that BUP strengthened the MDC fluorescence intensity in a concentration-related manner. These findings indicated the cells undergoing autophagy after BUP treatment.

LC3-II/I, p62 and Beclin-1 are autophagy marker proteins, and their expression levels were examined. Figure 7C,D showed that BUP upregulated Beclin1 expression levels, downregulated p62 expression levels, and promoted the transformation of LC3-I to LC3-II (the autophagosome-associated form) in A549 cells in a concentration-related manner.

### 3.5. RNA Sequencing

The numbers of raw reads in CK and BUP were 45,129,578 and 46,536,616, respectively. In total, 41.87 Gb of clean data was acquired, the percentage of Q30 base was above 92.63%. The clean reads were in comparison to the Trinity assembly data; the mapping results rate ranged from 94.81 to 95.94%. The results demonstrate that the mapped results and assembly integrity was good, and the data could be applied to the quantitative expression and annotation analyses, subsequently.

### 3.6. Differentially Expressed Genes (DEGs) in BUP-Treated Cells

**Figure 8:**
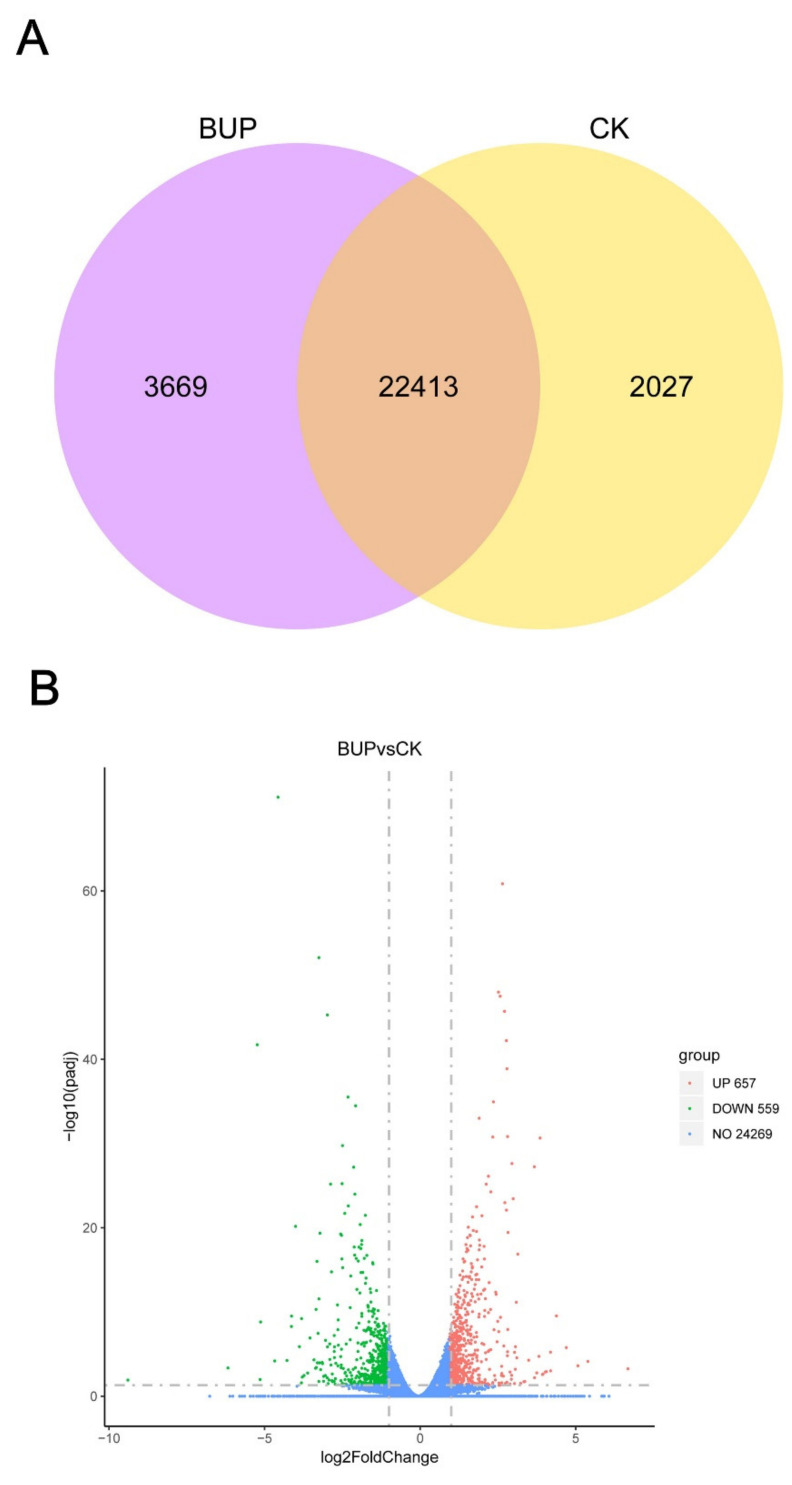
Analysis of expressed genes, showing: (**A**) Venn diagram of co-expressed and uniquely expressed genes between CK and BUP, (**B**) volcano map of DEGs. CK—control cells; BUP—BUP treated cells.

In this study, a total of 28,109 expressed genes were annotated. There were 22,413 (79.7%) genes expressed in both the control (CK) and treatment (BUP) groups, and 2027 (7.2%) and 3669 (13.1%) genes were particularly expressed in the CK and BUP groups, respectively (Figure 8A). Then, *p*-adjust < 0.05 and |log2FC| ≥ 1 were employed as screening criteria to determine the significant differences in gene expression. The results demonstrated that, in total, 1216 DEGs were acquired, among them were 657 upregulated genes and 559 downregulated genes (Figure 8B).

### 3.7. Expression of Related Genes after Treatment with BUP

**Figure 9:**
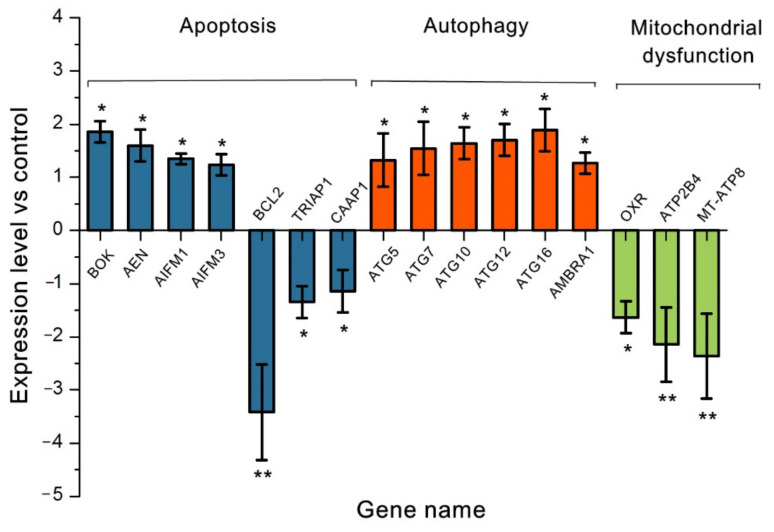
Expression variation of related genes at RNA transcription level after treatment with 80 μM BUP. Values are expressed as the mean and SEM values of three replicates. * *p* ≤ 0.05 and ** *p* ≤ 0.01 vs. the negative control.

The expression of apoptosis, autophagy, and mitochondrial damage-related genes at the RNA-transcription level was verified among the DEGs following 80 μM BUP treatment (Figure 9). Several apoptosis-related genes, e.g., BOK, AEN, AIFM1, and AIFM3, showed a significant increase. Moreover, BCL2, TRIAP1, and CAAP1 were significantly downregulated, by 3.41-, 1.34-, and 1.14-fold, compared with the control, respectively. The autophagy-related genes, e.g., ATG5, ATG7, ATG10, ATG12, ATG16, and AMBRA1, showed a significant increase, of 1.26- to 1.70-fold vs. control. In addition, antioxidant enzyme-related gene OXR, intracellular calcium homeostasis-related gene ATP2B4, and ATP synthesis-related gene MT-ATP8 were detected to be markedly downregulated.

### 3.8. GO Annotation and Enrichment Analyses of DEGs

**Figure 10:**
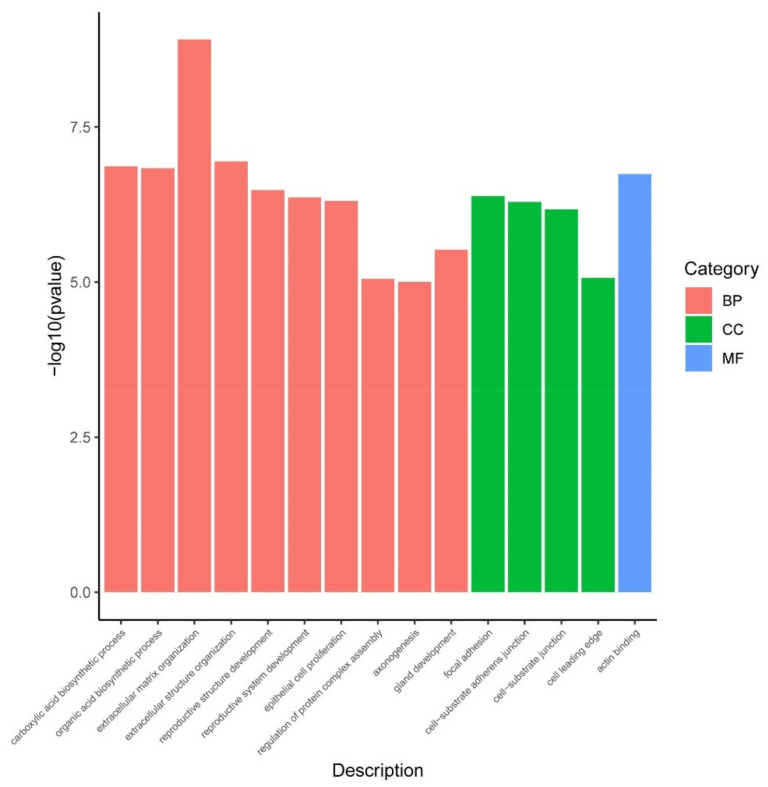
GO enrichment analysis of DEGs after BUP treatment.

GO functional annotation and enrichment analyses were performed on the DEGs. Then, the three kinds of annotations for the DEGs were categorized as molecular function (MF), cellular component (CC), and biological process (BP). Significant enrichment was found in 15 GO terms between the CK and BUP groups (Figure 10). The results showed that cellular substance synthesis and extracellular matrix were the most represented, with several enriched GO terms, such as “carboxylic acid biosynthetic process”, “organic acid biosynthetic process”, “extracellular matrix organization”, and “focal adhesion”.

### 3.9. KEGG Annotation and Enrichment Analyses of DEGs

**Figure 11:**
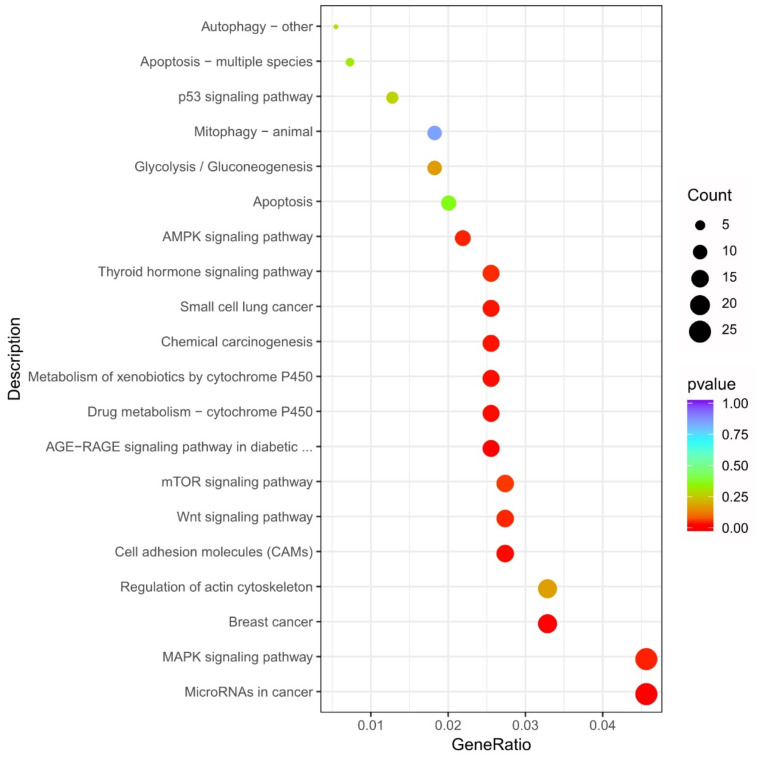
KEGG enrichment analysis of DEGs after BUP treatment.

To determine several related signaling pathways, the DEGs were identified using KEGG pathway enrichment analyses. In total, 20 pathways were enriched following BUP treatment (Figure 11). The results indicated that the cell death pattern and cell death-related signal pathways were the most represented, e.g., Apoptosis, Mitophagy–animal, Autophagy–other, p53 signaling pathway, AMPK signaling pathway, and mTOR signaling pathway. In addition, the differential genes were also enriched in the Chemical carcinogenesis, Metabolism of xenobiotics by cytochrome P450, and Drug metabolism-cytochrome P450 pathways. Cytochrome P450 enzymes are the main catalysts involved in the metabolism of drugs and are essential in the maintenance of general human health. Cytochrome P450s support the reductive, oxidative, and peroxidative metabolism of xenobiotic and endogenous substrates, such as agrochemicals, environmental pollutants, steroids, plant allelochemicals, fatty acids, and prostaglandins. These results strongly indicate the toxic effects of BUP on A549 cells and the cellular detoxification response to BUP treatment.

## 4. Discussion

Insecticides inhibiting chitin are a promising type of insect growth regulator because of their unique mechanism of action on pests and low toxicity to other species. Over the past few decades, the use of BUP has increased extensively. Although it is supposed to have low toxicity, its residues persist in the environment and might increase the latent health hazards for non-target organisms. For instance, the high levels of buprofezin present in the runoff from agricultural and industrial sources flow into the water, leading to the pollution of water environments [27,28]. However, previous information of BUP’s adverse effects on mammals, especially on human health, is limited. Therefore, in this study, the cytotoxicity of BUP to human cells was evaluated and the results showed BUP strongly inhibited viability of A549 cells, which suggests that BUP has latent toxicity to humans in vitro.

To further study the toxic mechanism of BUP on human cell lines, the mode of death of A549 cells was investigated. It was confirmed that BUP could trigger caspase-dependent apoptosis and autophagy associated with mitochondrial damage. Apoptosis, autophagy, and necrosis are well-known types of cell death, of which autophagy and apoptosis are particular forms of programmed cell death. These cell death processes can be induced via extensive irritation, such as growth factors, hormones, and pesticides. These processes are also regulated by the relevant gene network, which results in typical morphological changes and ultimately, cell death [29]. In this paper, through AV/PI double dyeing, we confirmed the occurrence of apoptosis in A549 cells after BUP treatment. In mammals, the apoptosis signaling is known to exist in two major pathways: the mitochondria-mediated intrinsic pathway and the cell death receptor-mediated extrinsic pathway. The release of cyt-c from the mitochondria is considered the earliest event and is one of the specific signs of mitochondrial pathway apoptosis. Firstly, the released cyt-c from the mitochondria into the cytoplasm induces Apaf-1 and drives the formation of apoptotic bodies, which contain pro-caspase-9. Consequently, caspase-9 leads to the activation of apoptosis effector caspase-3 and ultimately results in cell death [30]. The release of cyt-c is also regulated by the Bcl-2 protein family via controlling the permeability of mitochondrial membranes. This protein family is divided into pro-apoptotic proteins, such as Bax, and anti-apoptotic proteins, such as Bcl-2, according to function [31]. The pro-apoptotic protein Bax promotes cyt-c leakage to the cytoplasm, while the anti-apoptotic protein Bcl-2 inhibits cyt-c and prevents the fall of MMP [32]. In this paper, we confirmed that BUP-induced apoptosis in A549 cells occurs with the activation of caspases and the promotion ratio of Bax/Bcl-2 expression causing the leakage of cyt-c into the cytosol. The results indicate that BUP-induced mitochondria-mediated apoptosis pathways are involved in the caspase cascade.

Autophagy is an extremely conventional catabolic pathway that maintains cell homeostasis by degrading damaged or dysfunctional organelles and proteins. However, cell death could also be attributed to excessive autophagy [33]. In this paper, the formation of autophagosomes were detected by means of subcellular structure observation and fluorescence staining. The results confirmed the occurrence of autophagy after the treatment with BUP. A series of autophagy-related genes (ATG) and their encoded proteins play important roles in autophagy. Under the catalysis of ATG7 and ATG10, ATG12 and ATG5 are closely bound by an isopeptide and form the ATG12–ATG5 complex. Through the combination of non-covalent bonds, ATG5 further combines with the helix region of ATG16 to form the ATG12–ATG5–ATG16 complex, while ATG16 can form a larger complex by means of homo-oligomerization that is then bound to the pre-autophagosomal structure (PAS) to participate in its extension [34]. For autophagy-related protein, the conversion of LC3-I to LC3-II is acknowledged as a mark of autophagy. Once autophagy happens, LC3-I is transformed to LC3-II. Subsequently, LC3-II is recruited to the autophagosome membrane to promote the extension and maturation of the autophagosome [35]. Furthermore, p62 and Beclin-1 are both significant regulatory molecules that participate in autophagy. Beclin-1 is an essential component of nucleation of the autophagosome, and integrates into the pre-autophagosomal structure, playing an important role in autophagy initiation and progression [36]. A ubiquitin-binding domain and an LC3-interacting domain are included in p62, which acts as a selective autophagy receptor for the degradation of ubiquitinated substrates in autolysosomes [36]. When autophagy happens, p62 is also degraded. Our results exhibited that several autophagy-related genes, including ATG5, ATG7, ATG10, ATG12, and ATG16, were upregulated by BUP. We also observed that BUP increased the expression of Beclin1, as well as inhibiting p62 in A549 cells. These findings clearly prove that BUP induced autophagy in human cells from morphological and biochemical evidence.

Mitochondria are extremely sensitive and important in several cellular processes, including energy metabolism and programmed cell death. Mitochondrial dysfunction and damage can cause cell death based on their own membrane’s stability and calcium homeostasis [37]. Among the factors that contribute to mitochondrial injury, the extension of ROS is recognized as a main element. BUP treatment resulted in rapid ROS aggregation in A549 cells along with the decline of antioxidant enzyme-related gene expression, which demonstrated that BUP might induce oxidative stress in A549 cells through triggering ROS production and breaking antioxidant defenses. The oxidative stress further caused injury to mitochondrial membranes, eventually leading to mitochondrial damage and dysfunction. In this study, MMP collapse, as well as calcium homeostasis disorder were observed in BUP-treated A549 cells. In addition, BUP downregulated the MT-ATP8 gene that participates in encoding ATP synthase and is involved in mitochondrial ATP synthesis. The results demonstrated that excessive production of ROS had a further effect on the structure and function of the mitochondria. Moreover, the DEG enrichment analyses showed that a number of genes were identified in the AMPK and mTOR signaling pathways. AMP-activated protein kinase (AMPK) plays a crucial role in energy homeostasis and can be activated via ATP exhaustion. AMPK also acts as an inhibitor of the mammalian target of rapamycin (mTOR) that is a central regulator in controlling autophagy [38]. The results show the mechanism by which BUP may induce the blockage of energy metabolism in mitochondria and then induce autophagy in A549 cells via the AMPK-/mTOR-mediated pathway. Altogether, these results indicate that mitochondria might play an essential role in BUP-induced programmed cell death. Firstly, BUP induced mitochondrial apoptosis in human cells along with mitochondrial damage. Concurrently, the mitochondria damaged by oxidative stress were separately selected and eliminated through the autophagy process.

## 5. Conclusions

The findings of this study reveal that BUP could inhibit A549 cell viability by inducing mitochondria-mediated apoptosis and autophagy. The data reported here confirm the cytotoxity of BUP and further provide new insights into the activity of BUP in non-targeted human cells. This study highlights potential human health threats posed by BUP and may inform the treatment of BUP poisoning.

## Data Availability

Not applicable.
